# Evidence of Dual Molecular Diagnosis of a Young Male Patient With Hereditary Spastic Paraplegia Carrying Two Rare Autosomal Mutations

**DOI:** 10.7759/cureus.90036

**Published:** 2025-08-13

**Authors:** Yossy Machluf, Majd Said, Yigal Chechik, Shifra Ben-Dor, Yoram Chaiter

**Affiliations:** 1 Medical Corps, Israel Defense Forces, Tel Hashomer, ISR; 2 Department of Genomics, Shamir Research Institute, University of Haifa, Qatzrin, ISR; 3 Department of Life Sciences Core Facilities, Weizmann Institute of Science, Rehovot, ISR

**Keywords:** dstyk, dual molecular diagnosis, genetic variants, hereditary spastic paraplegia (hsp), neurodegenerative disorders, zfyve27

## Abstract

Hereditary spastic paraplegia (HSP) is a group of disorders that mostly affect the upper motor neurons of the spinal cord, with varying inheritance patterns and clinical presentations. We present the case of an 18-year-old male patient who was medically evaluated at a recruitment center due to non-specific complaints of joint pain and difficulty breathing over several years, without an established diagnosis or treatment. Physical examination revealed significant asymmetric muscle weakness in both legs and asymmetrical patellar reflexes. The patient was referred to diverse specialists and underwent a series of diagnostic tests, including blood work, serology, electromyography, nerve conduction tests, and magnetic resonance imaging of the brain and spine. No specific pathology related to the patient's symptoms was identified. Genetic testing, however, revealed heterozygous likely pathogenic variants in both *ZFYVE27* (c.898-2A>G) and *DSTYK* (c.1742T>C, p.Leu581Ser) genes, confirming the diagnosis of HSP. This case is notable for the rare occurrence of two distinct, very rare genetic mutations contributing to the pathogenesis of HSP, a situation called dual molecular diagnosis. We report a rare case of HSP dual molecular diagnosis. Both *DSTYK* and *ZFYVE27* act on membrane dynamics, critical in neuronal maintenance and axonal transport, essential processes disrupted in HSP. Further investigation is required to inform about potential interaction and the underlying molecular mechanism. Moreover, from the clinical perspective, the diagnostic workup brings to light the importance of awareness of a relatively rare disease and of thorough and comprehensive medical analysis to evaluate and rule out diverse potential causes.

## Introduction

Hereditary spastic paraplegia (HSP) is an umbrella term that embraces a clinically and genetically heterogeneous group of neurodegenerative disorders that affect the corticospinal tracts. HSP is classified into two subtypes based on phenotype: pure HSP (pHSP), which involves hallmark critical manifestations such as lower extremity bilateral progressive spasticity and triggered gait issues, hyperreflexia, muscle wasting and weakness, and extensor plantar responses, and complicated or complex HSP (cHSP), which includes more extensive neurological or non-neurological manifestations such as dementia, intellectual disability, epilepsy, optic atrophy, and peripheral neuropathy [1]. Symptoms of HSP can start at any age, from infancy to adulthood [2]. An inter- and intra-familial phenotype variability has been reported regarding age at onset, disease progression, the presence of associated neurological signs or disease severity, and implications.

HSP prevalence ranges from 0.1 to ~10 per 100,000 individuals [1,3,4], although higher rates have been documented in some specific regions and populations around the globe. The disorder can be inherited in various patterns: autosomal dominant, autosomal recessive, X-linked, mitochondrial inheritance, sporadic occurrence [5], and rare cases of patients with mosaic mutated variants have also been reported. Not only is HSP a rare condition, but the high clinical and genetic heterogeneity makes diagnosis challenging. To date, more than 80 susceptible gene loci associated with spastic gait disorders have been identified, involving more than 70 known causative spastic paraplegia genes [6-8]. The diagnostic rates of HSP are only 57%, 56%, and 21% for autosomal dominant, autosomal recessive, and sporadic HSP forms, respectively [9], suggesting that more genes are yet to be identified or that the current genetic diagnostic approaches are missing genetic alterations in regulatory and splicing regions [6]. Some of these mutations pose a fuzzy border between recessive and dominant inheritance, whereas some of the mutations overlap with other neurological conditions in terms of their clinical effects, highlighting the intricate connections between genetic, clinical, molecular, diagnostic, and pathophysiological aspects of the disorder [10].

## Case presentation

We present the case of an 18-year-old male patient who was medically evaluated at an Israel Defense Forces (IDF) regional recruitment center from January 2023 to April 2024 (For detailed information on the medical process at recruitment centers, see [11]). His medical history included minor iron deficiency anemia, joint pain since the age of 13, an adenoidectomy, and two years of breathing problems (normal spirometry in 2022). Kyphoscoliosis, deformity of toes 2 and 3 in his feet, asymmetric proximal muscular weakness in the lower extremities, particularly in the left leg, left-side patellar reflex asymmetry, and trouble getting out of a seated position were all discovered during the physical examination. On the other hand, the physical examination revealed no skin, hair, or craniofacial abnormalities. Except for color vision impairment, all other tests, such as anthropometric measurements, blood pressure, pulse, urinalysis, and eye tests, were within normal ranges.

**Figure 1:**
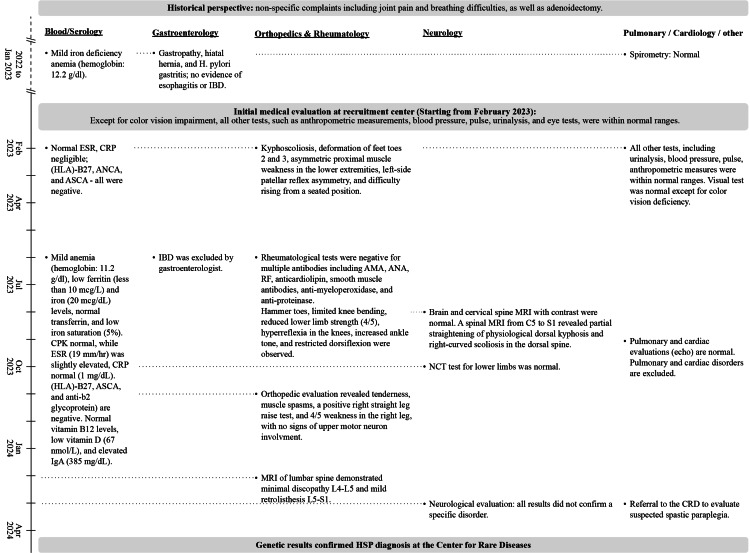
Schematic representation of the diagnostic workup that included the medical tests and findings in primary medical discipline along the chronological time axis. AMA: anti-mitochondrial antibodies; ANA: antinuclear antibody; ANCA: anti-neutrophil cytoplasmic antibody; ASCA: anti-Saccharomyces cerevisiae antibody; CPK: creatine phosphokinase; CRD: Center for Rare Diseases; CRP: C-reactive protein; EMG: electromyography; ESR: erythrocyte sedimentation rate; HLA-B27: human leukocyte antigen; IBD: inflammatory bowel disease; NCT: nerve conduction test; RF: rheumatoid factor.

During this period, spanning 16 months, the patient reported additional symptoms, including heel pain, muscle weakness, difficulties in standing, breathing issues, oral ulcers, a sense of jaw locking, and abdominal pain. The patient underwent a series of laboratory and diagnostic tests, including comprehensive blood work and serology. He was referred to evaluations and consultations with a rheumatologist, neurologist, pulmonologist, cardiologist, gastroenterologist, and orthopedic surgeon. Electromyography and nerve conduction tests for the lower limbs and MRI of the brain and cervical spine were conducted. Findings were mostly normal, and diverse diseases were ruled out. No specific pathology related to the patient's symptoms was identified. The whole diagnostic workup is described in Figure 1.

Even though HSP is a rare condition, it should be considered when no other obvious reason can be found for a patient's spasticity, stiffness, and neurological symptoms. It is often diagnosed by an exclusionary method, where genetic testing is the definitive final diagnostic step. The patient was sent to the Center for Rare Diseases to be evaluated for probable spastic paraplegia because of the enduring symptoms and spasticity despite normal test results.

The whole sequencing and genomic analysis were performed by the genomic center of CLALIT, the largest of Israel's four state-mandated health service organizations. A targeted gene panel was performed using next-generation sequencing (NGS) to detect mutations in genes known to be associated with HSP. Further bioinformatics analysis utilized either Franklin (Genoox) and/or Emedgene (Illumina) software. All are considered gold standard tools. 

**Figure 2:**
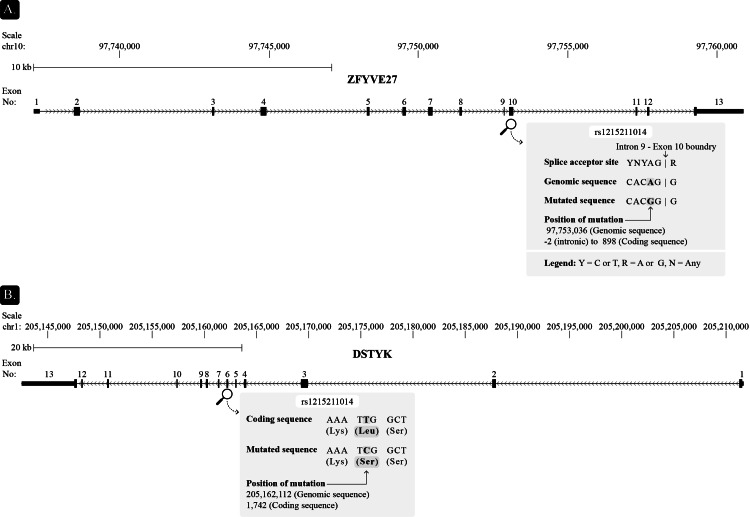
The genomic loci of (A) ZFYVE27 (GRCh38.p14:chr10:97,737,128-97,760,895) and (B) DSTYK (GRCh38.p14:chr1:205,142,505-205,211,702) separating mRNA coding (high black boxes) and untranslated (low black boxes) sequences, as well as the number and location of exons on the corresponding chromosomes. Gene orientation (on the + or - DNA strand) is shown by arrows on the line. Additionally, we provided an enlarged view of the mutations' site, sequence, and consequences.

**Figure 3:**
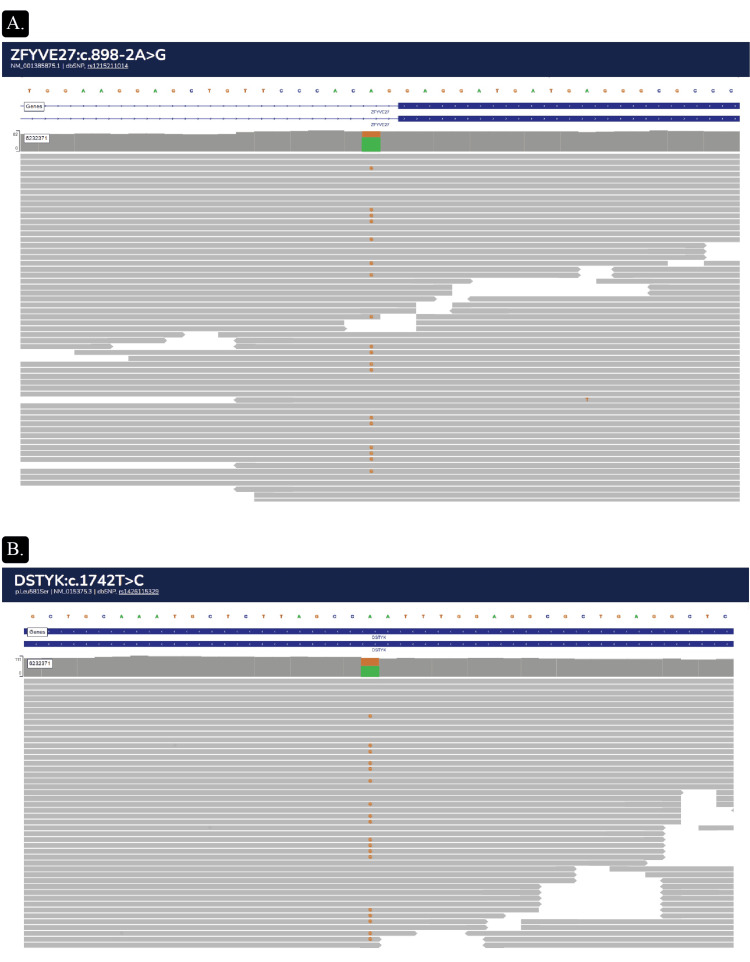
The genomic loci of (A) ZFYVE27 (GRCh38.p14:chr10:97,737,128-97,760,895) and (B) DSTYK (GRCh38.p14:chr1:205,142,505-205,211,702) separating mRNA coding (high black boxes) and untranslated (low black boxes) sequences, as well as the number and location of exons on the corresponding chromosomes. Gene orientation (on the + or - DNA strand) is shown by arrows on the line. Additionally, we provided an enlarged view of the mutations' site, sequence, and consequences.

Genetic testing revealed heterozygous mutations in two genes: *ZFYVE27 *(c.898-2A>G) and *DSTYK *(c.1742T>C, p.Leu581Ser) (Figure 2 and Figure 3). The mutations do not appear in the ClinVar database of human genetic variants and their relation to diseases (www.ncbi.nlm.nih.gov/clinvar/).

Nevertheless, both mutations are present in the Single Nucleotide Polymorphism Database (https://www.ncbi.nlm.nih.gov/snp). Both the ZFYVE27 (rs1215211014) and the DSTYK (rs1426115329) mutations are very rare, with a global allele frequency of 0.0000007 (1/1400302 GnomAD exomes) and 0.000004 (1/264690 TOPMED), respectively. Noteworthy, these genes were previously associated with HSP type 33 [12] (dominance inheritance) and 23 [13] (either dominance [13] or recessive [14] inheritance), respectively.

The ZFYVE27 A to G substitution disrupts a splice acceptor site (Figure 2A). Potentially, it may cause exon (10) skipping and a frameshift from this point, which is predicted to cause nonsense-mediated decay of the transcript. Alternatively, a cryptic splice site may be activated. As the mutation is located upstream of the sequence coding for the FYVE_protrudin domain, it could affect and even diminish the gene product function. The T to C substitution in DSTYK leads to a missense mutation (Figure 2B). Computational tools predict a deleterious effect on the gene product.

## Discussion

The persistence of neurological symptoms, despite initially normal results, prompted a thorough medical evaluation of an 18-year-old male patient as part of the military recruitment center's profiling process. This evaluation included detailed medical history, a physical examination, and various laboratory, imaging, and genetic tests. The results are highly suggestive of a rare HSP disease diagnosis. Furthermore, rare autosomal mutations were detected in two distinct HSP-associated genes, providing evidence of a rare HSP dual molecular diagnosis.

*ZFYVE27* (Gene ID: 118813) encodes a zinc finger FYVE-type containing 27 (also known as protrudin and SPG33), which is a known binding partner of myelin proteolipid protein 1 (SPG2), atlastin-1 (SPG3A), spastin (SPG4), reticulon 2 (SPG12), receptor expression-enhancing protein 1 and 5 (SPG31), Kif5A/B/C (SPG10), and other HSP-related proteins [12,15]. The protein protrudin interacts with tubular endoplasmic reticulum (ER)-shaping proteins and regulates ER morphogenesis and function [15]. *ZFYVE27* is an ER resident protein that promotes neurite formation through regulation of endosome trafficking [16].

*DSTYK* (Gene ID: 25778) encodes a dual serine/threonine and tyrosine protein kinase involved in cell death regulation, with multiple transcript variants producing different isoforms. Originally, a large 3' intragenic deletion in *DSTYK*, causing biallelic deletions/loss-of-function, was shown to underlie SPG23, an autosomal-recessive disorder characterized by spastic paraplegia and dyspigmentation [14]. Nevertheless, a recent exome analysis identified a novel, heterozygous dominant missense variant in *DSTYK* (c.271C>A (p.Leu91Met)) segregating with a familial SPG23 of mild lower-limb spasticity, voiding dysfunction, and seizures, supporting an autosomal dominant pattern for certain *DSTYK* mutations [13]. Additionally, mutations in *DSTYK* were shown to be associated with autosomal-dominant congenital malformations of the kidney and urinary tract [17,18].

Of note, genetic testing has not been conducted on the patient's family, as the extended core family, who are of North African heritage, including the parents, three daughters, and one son, has no notable related medical history or symptoms, so it is unclear whether the mutations were inherited or occurred de novo.

On the one hand, it is an uncommon discovery that the patient has rare mutations in two distinct genes, especially in the case of rare diseases. On the other hand, the identification of multiple rare variants in disease-associated genes is becoming increasingly common with the use of multi-gene panels and broad exome sequencing methods, especially for disorders with significant genetic heterogeneity involving dozens of disease-associated genes such as HSP. In fact, large-scale studies report that a notable proportion of patients undergoing broad sequencing, namely, 3% to 7% of patients with neurological disorders, present with more than one potentially relevant pathogenic or likely pathogenic variant contributing to the phenotype [19,20]. Therefore, in general, dual molecular diagnoses are not very common, but they are not extremely rare either and are more frequently observed in the context of rare and complex diseases, largely due to the broader use of exome/genome sequencing and the high rate of phenotypic heterogeneity.

While there is no evidence of direct or indirect interaction between *DSTYK *and* ZFYVE27*, both proteins act on membrane dynamics, critical in neuronal maintenance and axonal transport, essential processes disrupted in HSP. Therefore, carrying pathogenic variants in both genes might exert additive or synergistic effects on membrane trafficking, especially in long corticospinal neuron axons, potentially enhancing disease severity or onset. Further investigation is required to fully comprehend the clinical implications of these altered proteins and any potential connections between them.

## Conclusions

This case report demonstrates the importance of awareness of anamnestic and physical symptoms that should prompt consideration of HSP. We described a patient who exemplifies the diagnostic challenges where multiple negative test results are evident. Our patient carries exceedingly rare mutations in two different genes, *ZFYVE27 *and* DSTYK*, presenting a rare case of dual molecular diagnosis of HSP.
